# Bipolar investigation of near-surface glacial ice reveals an active microbial ecosystem driven by photosynthesis and chemolithoautotrophy

**DOI:** 10.1093/ismeco/ycag105

**Published:** 2026-04-22

**Authors:** Brady R W O’Connor, Donovan Allen, Matthew Quinn, Melissa Kozey, Richard J Léveillé, Lyle G Whyte

**Affiliations:** Department of Natural Resource Sciences/McGill Space Institute, McGill University, Macdonald Campus, 21111 Lakeshore Road, Sainte-Anne-de-Bellevue, Quebec H9X 3V9, Canada; Department of Natural Resource Sciences/McGill Space Institute, McGill University, Macdonald Campus, 21111 Lakeshore Road, Sainte-Anne-de-Bellevue, Quebec H9X 3V9, Canada; Department of Natural Resource Sciences/McGill Space Institute, McGill University, Macdonald Campus, 21111 Lakeshore Road, Sainte-Anne-de-Bellevue, Quebec H9X 3V9, Canada; Department of Natural Resource Sciences/McGill Space Institute, McGill University, Macdonald Campus, 21111 Lakeshore Road, Sainte-Anne-de-Bellevue, Quebec H9X 3V9, Canada; Department of Earth and Planetary Sciences, McGill University, 845 Sherbrooke Street West, Montreal, Quebec H3A 0G4, Canada; Department of Natural Resource Sciences/McGill Space Institute, McGill University, Macdonald Campus, 21111 Lakeshore Road, Sainte-Anne-de-Bellevue, Quebec H9X 3V9, Canada

**Keywords:** glaciers, glacial microbiology, microbial ecology, cryobiology, astrobiology, Arctic, Antarctic, ice

## Abstract

Despite extreme conditions including freezing temperatures, low water activity, and few nutrients, active microorganisms are thought to inhabit glacial ice, yet little is known about their identities and methods of survival. We used flow cytometry, cultivation, metagenomics, and metatranscriptomics to characterize viable and active microbial communities from near-surface englacial ice from White Glacier in the Canadian High Arctic and Johnsons Glacier on Livingston Island, Antarctica. The ice, though low in microbial biomass (10^4^ cells/ml), harbors communities capable of growth at subzero temperatures (−5°C), high salinity (12% NaCl), and low pH (pH 3). The communities of both poles were different, with metagenome-assembled genomes (MAGs) from White Glacier belonging to *Cyanobacteriota* and novel phyla and MAGs from Johnsons Glacier belonging to *Pseudomonadota* and *Actinomycetota*. Despite this, both glacial communities shared key metabolic functions, including aerobic respiration, aerobic carbon monoxide oxidation, sulfide oxidation, and denitrification. Metatranscriptomics from White Glacier revealed dominant *Cyanobacteriota*, performing oxygenic photosynthesis and carbon fixation and accompanied by active lithoautotrophs performing metabolisms such as carbon fixation via the 3-hydroxyproprionate cycle, anoxygenic photosynthesis, sulfide oxidation, and nitrate reduction/denitrification. These metabolisms appear to support an active heterotrophic community performing aerobic respiration and aerobic carbon monoxide oxidation. This study highlights the distinct but functionally similar microbial communities in Arctic and Antarctic glaciers, hinting that there may be a core set of metabolisms required for surviving in englacial ice and suggesting that similar communities could persist in glacial ice on Mars or the icy outer moons, Europa and Enceladus.

## Introduction

Climate change is disproportionately impacting Earth’s polar regions. The warming rate in the Arctic is almost three times faster than the global average of 0.6°C–0.75°C per decade [1, 2]. On the contrary, Antarctica is warming at a slower rate (0.31°C per decade) [3] but is expected to accelerate in the coming decades [4]. Given this, glaciers are losing mass at startling rates; combined, Greenland and the Canadian Arctic are losing 66 gigatons annually, while Antarctica is losing 20.9 gigatons annually [5]. This retreat poses a threat to one of the worst understood microbial ecosystems on Earth: those that are entrapped within glacial ice.

Between 10^25^ and 10^29^ microorganisms are contained within glaciers globally, with 10^17^ to 10^21^ of those discharged annually through melt [6, 7]; however, this figure is anticipated to rise as warming intensifies. The discharge of these cells has the potential to change downstream ecosystems through the alteration of soil development, nutrient cycling, and productivity in both terrestrial and marine environments. While supra and subglacial habitats provide the bulk of our knowledge about these microorganisms, englacial ice, which contains comparable cell abundances by total volume, provides very little [8].

Englacial ice, defined as glacier ice not directly influenced by surface or subglacial processes [9], has long been considered too extreme to support more than maintenance metabolism [10]. Subfreezing temperatures, low water activity, and low nutrient concentrations make survival particularly challenging. Nevertheless, microorganisms have been detected in englacial ice globally using both culture dependent [11–16] and independent approaches [17–19], indicating that these environments may host metabolically active communities. Simon *et al*. reported the first englacial metagenome, revealing mainly aerobic heterotrophs alongside pathways for autotrophic carbon fixation and nitrite/nitrate reduction but without evidence of *in situ* activity [20]. Evidence from laboratory cultures and irregularities in ice core gas records (CH_4_ and N_2_O) accompanied by spikes in taxa known to produce those gasses did provide evidence that englacial microbes are capable of metabolism *in situ* [21–27]. However, direct evidence of active microbial communities *in situ*—and the molecular strategies that enable survival under these extreme conditions, such as those obtained via metatranscriptomics—has remained elusive.

Obtaining a better understanding of englacial microbial survival is important not only for predicting their influence on Earth’s ecosystems as glaciers retreat, but also for astrobiology. Ice-rich environments exist on Mars and the icy moons of the outer solar system [28, 29]. If life ever evolved on these celestial bodies, it may persist today close to the surface and relatively accessible for future life detection missions [30]. Studying microbial survival strategies in cold, low-nutrient, and high-stress englacial environments on Earth therefore provides valuable analogs for the search for life in extraterrestrial ice.

Here, we combine metagenomics and metatranscriptomics with flow cytometry and cultivation to test whether Arctic and Antarctic near-surface glacial ice hosts viable, active microbial communities and to characterize their metabolic and adaptive strategies *in situ*. To our knowledge, this work presents the first metatranscriptome ever recovered from englacial ice. Together, these datasets provide what we believe is the highest-resolution view of the taxonomic composition, functional potential, and *in situ* activity of microbes entrapped within glacial ice.

## Materials and methods

Detailed descriptions of the methods are in Supplementary Materials.

### Sampling site descriptions and sample collection

**Figure 1:**
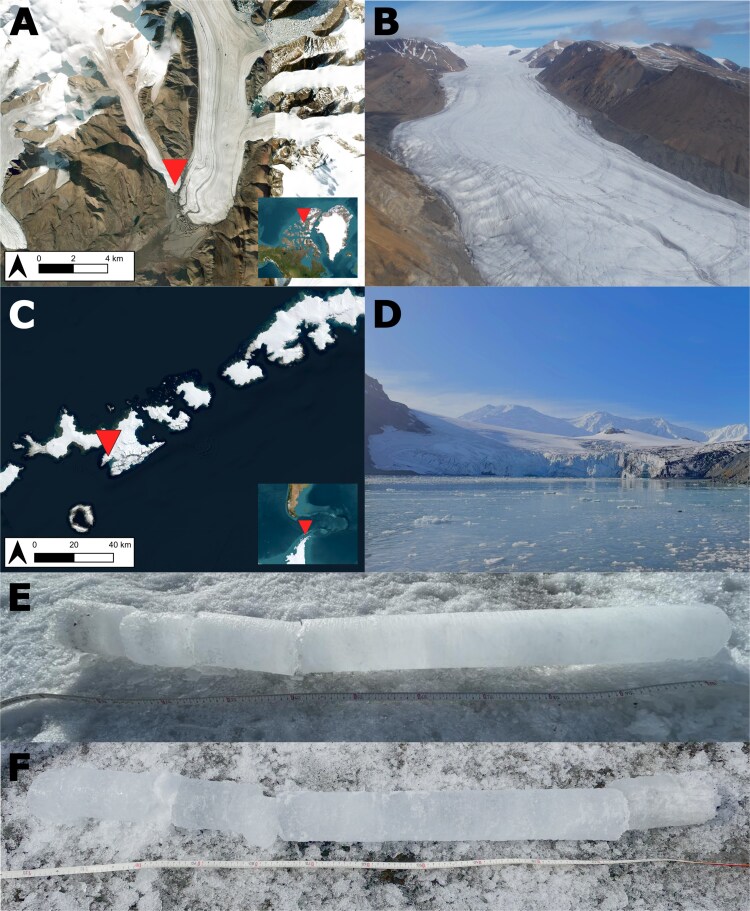
(A) Location of White Glacier on Axel Heiberg Island in Nunavut, Canada (Photo credit: Environmental Systems Research Institute); (B) arial view of White Glacier (Photo credit: Scott Sugden); (C) location of Johnsons Glacier on Livingston Island, Antarctica (Photo credit: Environmental Systems Research Institute); (D) photo of Johnsons Glacier; (E) one of the White Glacier cores used in this study; (F) one of the Johnsons Glacier cores used in this study.

We traveled to White Glacier (79.443163N, 90.639496W) in July 2022 and 2023 and Johnsons Glacier (62.66925S 60.63998W) in February 2023. Surface ice cores were collected to a maximum depth of 1.5 m (Fig. 1) before being transported frozen to the laboratory at McGill University. From White Glacier, a core subsection of 0.7–0.9 m was chosen for further study, while from Johnsons Glacier, a subsection of 1–1.2 m was chosen. Light and temperature readings were recorded from inside the borehole immediately after collecting the cores. The concentration of nutrients and metals was performed by the Natural Resources Analytical Laboratory at the University of Alberta (Edmonton, Alberta, Canada).

### Core decontamination and processing

An artificial core was constructed as a negative control and processed the same way as the sample cores. To decontaminate the exterior of the cores, we adapted a method described by Coelho *et al*. [31]. The core sections were allowed to melt directly into DNA/RNA Shield reagent at 4°C in the dark to prevent microbial metabolism during ice thaw. Thawed core samples were filtered to concentrate cell material for DNA/RNA extraction and sequencing.

### DNA/RNA extraction and sequencing

DNA and RNA were extracted and purified from DNA/RNA-free water, filtered artificial ice, and ice core samples. RNA was treated to remove extracted carryover DNA, and then ribosomal RNA was depleted, and complimentary DNA (cDNA) was generated. Sequencing was performed at The Centre for Applied Genomics (Toronto, Ontario, Canada), with nucleic acids recovered from White Glacier being sequenced on a NovaSeq 6000 (Illumina, San Diego, California), while those from Johnsons Glacier were sequenced on a NovaSeq X (Illumina, San Diego, California). RNA sequencing was not successful from the Johnsons Glacier core.

### Metagenome and metatranscriptome data analysis

Metagenome and metatranscriptome data analysis largely followed the methods used by Magnuson *et al*. [32] with some modifications. Trimmomatic was used to trim reads [33]. Negative control reads were removed from the samples with DeconSeq [34]. Decontaminated metagenome and metatranscriptome reads were classified with Kaiju through the KBase platform [35, 36], and the metagenome reads were assembled with MegaHit [37]. Assembled metagenome contigs were binned with MetaBat2 [38], MaxBin2 [39], and SemiBin2 [40]. The bins were dereplicated with dRep [41]. The quality of each bin was checked with CheckM2 [42] and taxonomically identified with Genome Taxonomy Database Toolkit (GTDB-tk) [43]. iRep [44] was used to estimate bin replication rate.

Contaminating ribosomal RNA sequences were removed from the metatranscriptome with SortMeRNA [45], and contaminant human reads were removed with the removehuman tool (Bushnell B. – sourceforge.net/projects/bbmap/). The metatranscriptomes were aligned to the metagenomes using bowtie2 [46] and counted using HTSeq2 [47]. Functional annotation was performed using the JGI IMG/M annotation pipeline [48].

All bioinformatic workflows, software versions, and command-line parameters used in this study are publicly available at: https://github.com/anxious-astrobiologist/Bipolar-englacial-bioinformatics-pipeline.

### Ice core cell isolation and characterization

From each glacier, a replicate core from the same depth used for sequencing was decontaminated in the same way as the cores used for nucleic acid sequencing, and 500 ml was melted at 4°C in the dark. Melted, filtered ice was resuspended, plated and/or inoculated into various aerobic media types used by Miteva and Brenchley [49], and incubated at 5°C. Isolated colonies were tested for growth in a range of salt, pH, and temperature conditions. DNA extracts of some isolates were sent to the Plate-forme d’Analyses Genomiques de l’Université Laval (Quebec City, Quebec, Canada) for 16S ribosomal Ribonucleic Acid sequencing to determine their identity. A random selection of isolates also had their metabolism characterized at −2°C using Biolog Gen III plates.

### Flow cytometry

To quantify the total and live cell concentration in the ice cores, an aliquot of resuspended cells in Phosphate-Buffered Saline (PBS) was live/dead stained using the LIVE/DEAD™ BacLight™ Bacterial Viability Kit (Invitrogen, Waltham, Massachusetts) following the manufacturer’s instructions. Flow cytometry measurements were conducted on a Guava easyCyte (Millipore, Burlington, Massachusetts). The sample was subjected to 5000 events, with the concentration of viable bacteria being determined by deducting the blank values.

## Results/discussion

### The microbial communities of White and Johnsons glaciers are taxonomically distinct

**Figure 2:**
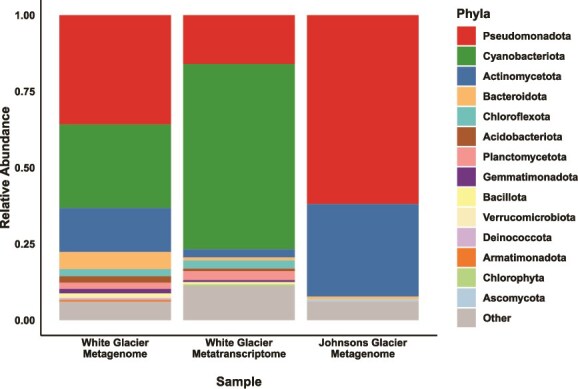
Taxonomic composition of the metagenomes and metatranscriptome from White and Johnsons Glacier; the estimation was performed on quality filtered and decontaminated unassembled reads; phyla with a relative abundance below 0.5% were grouped into “Other.”

To identify the taxonomic composition of the microbial communities in both glaciers, we used Kaiju, which revealed both shared and distinct communities. Several cold-adapted taxa were abundant in both systems, including *Glaciihabitans* sp. INWTZ (1.7% and 4.3% of White and Johnsons Glaciers, respectively) and *Frigoribacterium* sp. CG_9.8. *Phormidesmis priestleyi*, the most abundant species in White Glacier (6.2%), was also detected in Johnsons Glacier at a lower abundance. Despite these shared taxa, the glaciers differed markedly at the phylum level. White Glacier was dominated by *Pseudomonadota* (36%), *Cyanobacteriota* (27%), *Actinomycetota* (14%), and *Bacteroidota* (6%), whereas Johnsons Glacier was dominated by *Pseudomonadota* (62%) and *Actinomycetota* (30%) (Fig. 2).

**Figure 3:**
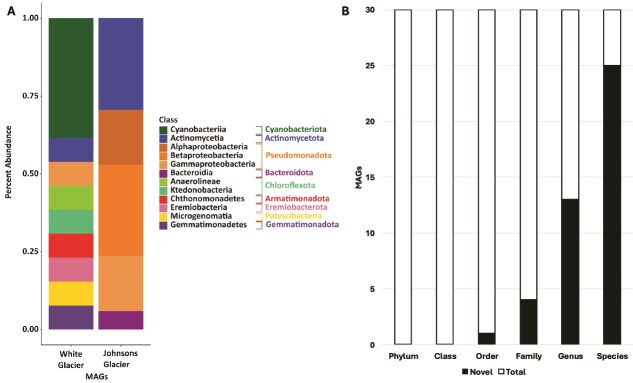
Metagenome assembled genome taxonomy and novelty; (A) MAGs composition from each glacier listed by class and phylum; (B) taxonomic novelty of the MAGs recovered in this work; the degree of novelty was determined based on the lowest taxonomic rank with which GTDB-tk could assign a recognized taxon name.

Thirty metagenome-assembled genomes (MAGs) were recovered, including 13 from White Glacier and 17 from Johnsons Glacier (Table 1). Consistent with metagenomic patterns, White Glacier MAGs were more taxonomically diverse and included five *Cyanobacteriota* genomes, as well as representatives of rare or recently described phyla (*Armatimonadota, Eremiobacterota, Patescibacteria*, and *Gemmatimonadota*) (Fig. 3). In contrast, Johnsons Glacier MAGs were largely affiliated with *Pseudomonadota* and *Actinomycetota*. No MAGs were shared between glaciers. Across both datasets, GTDB classification indicated substantial novelty, with 25 MAGs novel at the species level, 13 at the genus level, 4 at the family level, and 1 White Glacier MAG (WG MAG 8; *Anaerolineae*), representing a putative novel order (Fig. 3). Given the limited availability of genomes from polar glacial ice [18], these MAGs provide an important resource for investigating microbial survival strategies and the functional potential of poorly characterized glacial taxa.

**Table 1:** Identities and statistics of medium and high-quality MAGs recovered from White and Johnsons Glaciers.

	Name	Completeness	Contamination	Classification	TPM
White Glacier	MAG 1	92.92	3.98	p__*Actinomycetota*;c__*Actinomycetia*;o__*Actinomycetales*;f__*Microbacteriaceae*;g__*Lacisediminihabitans*	7688
	MAG 2	54.37	0.71	p__Cyanobacteria;c__*Cyanobacteriia*;o__*Cyanobacteriales*;f__*Microcoleaceae*;g__*Microcoleus*	142 735
	MAG 3	58.44	6.91	p__Cyanobacteria;c__*Cyanobacteriia*;o__*Cyanobacteriales*;f__*Microcoleaceae*;g__*Microcoleus*	63 203
	MAG 4	82.17	7.94	p__*Armatimonadota*;c__*Chthonomonadetes*;o__*Chthonomonadales*;f__*Chthonomonadaceae*	6618
	MAG 5	61.74	6.26	p__*Eremiobacterota*;c__*Eremiobacteria*;o__Baltobacterales;f__*Baltobacteraceae*	381
	MAG 6	99.65	0.95	p__Cyanobacteria;c__*Cyanobacteriia*;o__*Cyanobacteriales*;f__*Coleofasciculaceae*;g__PCC7113	51 949
	MAG 7	93.88	3.17	p__*Patescibacteria*;c__*Microgenomatia*;o__*Levybacterales*;f__UBA12049;g__PPGL01	127
	MAG 8	69.56	8.69	p__*Chloroflexota*;c__*Anaerolineae*;o__SBR1031;f__UBA2796	22 131
	MAG 9	55.82	1.73	p__*Gemmatimonadota*;c__*Gemmatimonadetes*;o__*Gemmatimonadales*;f__*Gemmatimonadaceae*;g__*Gemmatimonas*	3824
	MAG 10	85.86	4.24	p__*Chloroflexota*;c__*Ktedonobacteria*;o__Ktedonobacterales;f__*Ktedonobacteraceae*;g__DTNP01	75 95
	MAG 11	69.38	7.79	p__*Pseudomonadota*;c__*Gammaproteobacteria*;o__*Steroidobacterales*;f__*Steroidobacteraceae*;g__Bog-1198	27 01
	MAG 12	57.56	9.75	p__Cyanobacteria;c__*Cyanobacteriia*;o__*Cyanobacteriales*;f__*Nostocaceae*	18 681
	MAG 13	75.40	0.87	p__Cyanobacteria;c__*Cyanobacteriia*;o__*Leptolyngbyales*;f__*Leptolyngbyaceae*;g__*Phormidesmis*_A;s__*Phormidesmis*_*A priestleyi*_B	3527
Johnsons Glacier	MAG 1	98.40	0.99	p__*Actinomycetota*;c__*Actinomycetia*;o__*Mycobacteriales*;f__*Mycobacteriaceae*;g__X156	N/A
	MAG 2	99.99	1.09	p__*Pseudomonadota*;c__*Alphaproteobacteria*;o__*Sphingomonadales*;f__*Sphingomonadaceae*;g__*Sphingomonas*;s__*Sphingomonas aquatilis*	N/A
	MAG 3	97.37	1.65	p__*Actinomycetota*;c__*Actinomycetia*;o__*Mycobacteriales*;f__*Pseudonocardiaceae*;g__*Pseudonocardia*	N/A
	MAG 4	99.75	5.56	p__*Pseudomonadota*;c__*Betaproteobacteria*;o__*Burkholderiales*;f__*Burkholderiaceae*;g__*Paraburkholderia*;s__*Paraburkholderia* sp005503145	N/A
	MAG 5	95.61	2.29	p__*Pseudomonadota*;c__*Gammaproteobacteria*;o__*Xanthomonadales*;f__*Xanthomonadaceae*;g__*Stenotrophomonas*;s__*Stenotrophomonas maltophilia*_AM	N/A
	MAG 6	77.22	6.14	p__*Pseudomonadota*;c__*Betaproteobacteria*;o__*Burkholderiales*;f__*Burkholderiaceae*;g__*Rhodoferax*	N/A
	MAG 7	79.25	9.41	p__*Pseudomonadota*;c__*Gammaproteobacteria*;o__*Xanthomonadales*;f__*Rhodanobacteraceae*;g__*Rhodanobacter*	N/A
	MAG 8	83.94	4.50	p__*Pseudomonadota*;c__*Gammaproteobacteria*;o__*Pseudomonadales*;f__*Pseudomonadaceae*;g__*Pseudomonas*_E	N/A
	MAG 9	89.72	4.58	p__*Pseudomonadota*;c__*Betaproteobacteria*;o__*Burkholderiales*;f__*Burkholderiaceae*;g__*Polaromonas*	N/A
	MAG 10	100	4.62	p__*Actinomycetota*;c__*Actinomycetia*;o__*Mycobacteriales*;f__SCTD01	N/A
	MAG 11	76.80	2.00	p__*Actinomycetota*;c__*Actinomycetia*;o__*Mycobacteriales*;f__SCTD01	N/A
	MAG 12	89.31	4.72	p__*Actinomycetota*;c__*Actinomycetia*;o__*Actinomycetales*;f__*Dermatophilaceae*;g__UBA4719	N/A
	MAG 13	98.60	0.49	p__*Pseudomonadota*;c__*Alphaproteobacteria*;o__*Acetobacterales*;f__*Acetobacteraceae*;g__CAITOL01	N/A
	MAG 14	92.89	1.53	p__*Bacteroidota*;c__*Bacteroidia*;o__*Sphingobacteriales*;f__*Sphingobacteriaceae*;g__*Pelobium*;s__	N/A
	MAG 15	81.10	1.64	p__*Pseudomonadota*;c__*Betaproteobacteria*;o__*Burkholderiales*;f__*Burkholderiaceae*;g__*Paraburkholderia*;s__*Paraburkholderia fungorum*	N/A
	MAG 16	100	3.96	p__*Pseudomonadota*;c__*Alphaproteobacteria*;o__*Rhizobiales*;f__*Xanthobacteraceae*;g__*Bradyrhizobium*;s__*Bradyrhizobium* sp003020075	N/A
	MAG 17	90.58	1.83	p__*Pseudomonadota*;c__*Betaproteobacteria*;o__*Burkholderiales*;f__*Burkholderiaceae*;g__*Cupriavidus*;s__*Cupriavidus pauculus*	N/A

Completeness and contamination levels were estimated with CheckM2, and taxonomy was determined using GTDB-tk. TPM aligned to each MAG from White Glacier are listed. No metatranscriptome was recovered from Johnsons Glacier, and thus, transcripts could not be mapped to the MAGs recovered from this site. N/A = not applicable.

Lower taxonomic diversity observed in Johnsons Glacier could reflect differences in sequencing depth; however, this is unlikely. The Johnsons Glacier metagenome contained more reads, produced better assembly statistics, and yielded more MAGs than White Glacier (Supplemental File), indicating better coverage. Instead, community differences likely reflect contrasting source environments, particularly for phototrophs. White Glacier is land-terminating, bordered by snow-free terrain that promotes eolian input of terrestrial microorganisms, and is covered by cryoconite holes dominated by *Cyanobacteriota* that act as local phototroph reservoirs [50–52]. In contrast, Johnsons Glacier is sea-terminating and surrounded by largely snow-covered landscapes, likely receiving greater marine aerosol and precipitation inputs that are typically less enriched in *Cyanobacteriota* [53]. These factors likely account for the higher relative abundance of *Cyanobacteriota* and greater taxonomic diversity in White Glacier.

### Aerobic and anaerobic metabolisms are present and active in polar glacial ice

**Figure 4:**
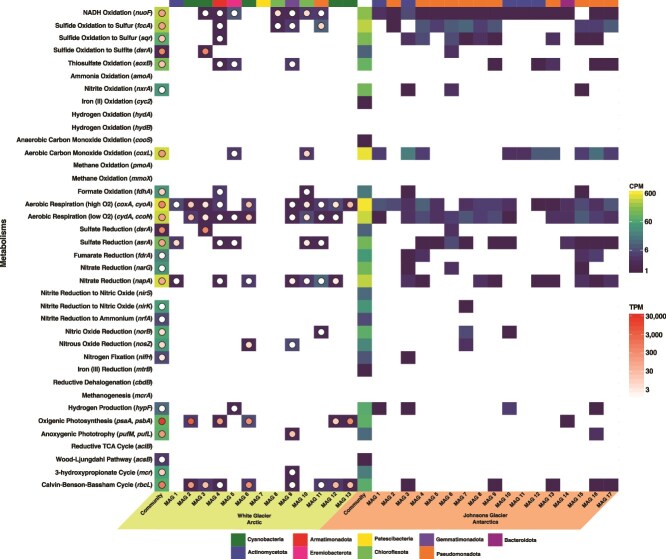
Composition of metabolic marker genes from the metagenomes, metatranscriptome, and MAGs of White Glacier and Johnsons Glacier; genes in the metagenome and MAGs are represented by the square heatmap and expressed as CPM; the metatranscriptome aligned to the White Glacier metagenome and MAGs is represented by the circle heatmap overlaying the squares and is expressed as TPM.

To determine how glacially entrapped microbial communities might be adapted to the ice environment, we screened the glacial metagenomes for marker genes representing 38 diverse metabolisms (Fig. 4). Broadly, metabolic similarities exist between the glaciers, suggesting a common survival mode despite their few taxonomic similarities and geographic isolation.

**Figure 5:**
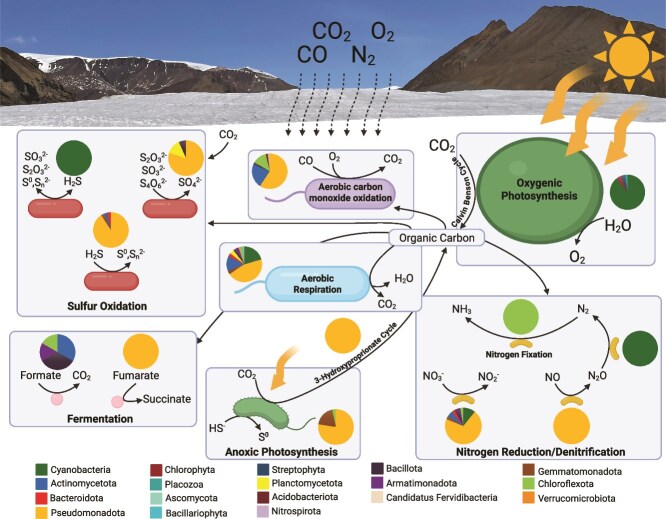
Summary of active metabolisms based on metabolic marker gene transcripts found in White Glacier; phyla performing each metabolism are marked by the pie charts; the size of each of the microorganisms reflects the relative transcript abundance of each metabolism and is not intended to represent quantitative differences in metabolic rates; created in BioRender. O’Connor, B. (2026) https://BioRender.com/vmkmdge

Aerobic metabolisms were abundant within the metagenomes of both glaciers. The organic carbon concentration measured in the melted ice was 4.05 mg/l and 2.17 mg/l in White Glacier and Johnsons Glacier, respectively (Table 2). The *coxA/cyoA* genes, responsible for aerobic respiration in high oxygen environments, were two of the most abundant marker genes we surveyed [White Glacier: 450.96 copies per million genes (cpm); Johnsons Glacier: 554.03 cpm], followed by the *cydA/ccoN* genes (White Glacier: 346.01 cpm; Johnsons Glacier: 353.01 cpm), responsible for aerobic respiration in low oxygen environments (Fig. 4). Both sets were also present in most MAGs recovered from both glaciers. Within White Glacier, *CoxA/CyoA* and *CydA/CcoN* genes were highly expressed [526.59 transcripts per million transcripts (tpm) and 113.89 tpm, respectively] in the metatranscriptome (Fig. 4) and attributed mainly to *Pseudomonadota, Cyanobacteriota*, and *Actinomycetota*, but also to nine other phyla, indicating that a diverse aerobic microbial community is metabolically active within the 1 m englacial ice environment of White Glacier (Fig. 5).

**Table 2:** Cell abundance and physicochemical characteristics of White Glacier and Johnsons Glacier ice cores.

Parameter	White Glacier	Johnsons Glacier
Total cell abundance (cells/ml)	4.75 × 10^4^	ND
Live fraction	0.50%	ND
Ice temperature (°C –sample depth)	−0.2	−0.1
Total organic carbon (mg/l)	4.05	2.17
Total inorganic carbon (mg/l)	0.602	0.856
NH_4_^+^ (μg/l)	36.432	15.498
NO_2_^−^ (μg/l)	1.774	<LOQ
NO_3_^−^ (μg/l)	133.676	<LOQ
PO_4_^3−^ (μg/l)	<LOQ	<LOQ
SO_4_^2−^ (mg/l)	<LOQ	<LOQ
Total S (mg/l)	0.245	0.059
Cl^−^ (mg/l)	0.656	0.42
EC (μS/cm)	13.1	3.97
pH	6.96	5.59

LOQ = limit of quantification, ND = no data

Interestingly, *coxL*, responsible for aerobic carbon monoxide oxidation, was the fourth most abundant marker gene we surveyed from the White Glacier metagenome (377.17 cpm) and was the second most abundant in Johnsons Glacier (486.62 cpm) (Fig. 4). *CoxL* transcripts were expressed in White Glacier (160.19 tpm) (Fig. 4), predominantly by *Pseudomonadota*, as well as *Actinomycetota* and *Chloroflexota* (Fig. 5). Further, *coxL* was found in MAGs from both glaciers including WG MAG 10 (*Ktedonobacteraceae*) and WG MAG 5 (*Baltobacteraceae*), a member of the under-represented and newly recognized *Eremiobacterota* phylum, making this the first evidence for aerobic CO oxidation by this phylum and expanding its metabolic versatility. In Johnsons Glacier, *coxL* was also present in 10 MAGs, 5 of which also encoded Calvin–Benson markers, indicating the genomic potential for chemolithoautotrophic CO oxidation (Fig. 4). However, other than JG MAG 3 (*Pseudonocardia*), lithoautotrophic CO oxidation has not been demonstrated in any of the corresponding genera [54, 55], and given the low CO concentrations expected in glacial ice, CO oxidation in both glaciers is most likely heterotrophic as has previously been shown as a survival mechanism during periods of starvation [55–58].

While aerobic metabolisms were predominant, within both glacier metagenomes, fermentative metabolisms were also present, including fumarate reduction. The *frdA* marker gene was present in both glaciers (White Glacier: 19.68 cpm; Johnsons Glacier: 40.45 cpm) but only in MAGs from Johnsons Glacier (Fig. 4). Formate oxidation is another form of anaerobic metabolism, which was also present in both glaciers (White Glacier: 34.44 cpm; Johnsons Glacier: 15.93 cpm) and some MAGs (Fig. 4).

The coexistence of oxic and anoxic metabolisms in near-surface glacial ice indicates significant microscale heterogeneity. Trapped atmospheric gasses (10%–15% by volume) supply oxygen, carbon monoxide, and carbon dioxide [59–61] to sustain aerobic metabolism. However, slow diffusion through ice and the structuring of gas concentrations by bubble distribution, ice crystal boundaries, liquid veins, and local microbial consumption result in spatial variation [23, 62, 63]. Ice core research shows that oxygen can be rapidly depleted in liquid veins and cryoconcentrated brines, generating microaerophilic or anoxic niches within otherwise oxic ice [64, 65]. Sequencing surveys also reveal the coexistence of aerobic taxa with facultative and obligate anaerobes [20, 27], supporting spatially heterogeneous microenvironments. The presence of high-oxygen terminal oxidases (*coxA/cyoA*), low-oxygen respiratory pathways (*cydA/ccoN*), and anaerobic metabolisms in both glaciers likely reflects adaptation to microscale oxygen variability.

### Photo and autotrophic metabolisms are present and active in polar near-surface ice

We hypothesized that in the summer, sunlight penetrating the near-surface of glacial ice could provide a source of energy to a microbial ecosystem. Light penetration was measured in White Glacier on a clear, sunny day in July 2023 to evaluate whether the light levels at the sampling depth were adequate to support photosynthesis. In White Glacier, light intensity measurements revealed that 3.2% of surface light reached the sampling depth, equivalent to a Photosynthetically Active Radiation (PAR) of 48.1 μmol photons m^−2^ s^−1^, which is sufficient to sustain photosynthesis [66, 67].

To determine if near-surface glacial microbial communities were exploiting this energy source, we searched the metagenomes of both glaciers for genes associated with photosynthesis and autotrophic carbon fixation. Genes for anoxygenic and oxygenic photosynthesis were found in both glaciers. Both sites contain *psaA* and *psbA*, which encode marker proteins for Photosystems I and II (White: 82 cpm; Johnsons: 74.8 cpm). Within White Glacier, 56 of the 66 proteins needed for oxygenic photosynthesis were found using the Kyoto Encyclopedia of Genes and Genomes database reconstruction tool, indicating the existence of a whole pathway. White Glacier also contained many genes for anoxygenic photosynthesis (*pufM, pufL*) (72.15 cpm), whereas Johnsons Glacier had much fewer (22.06 cpm) (Fig. 4).

The White Glacier metatranscriptome confirmed that photosynthesis was highly active: *PsaA* and *PsbA* were the most abundant transcripts (20 789.5 tpm), accounting for 3.5% of all reads. These were almost exclusively attributed to *Cyanobacteriota*, which dominated the active community (62% of all transcripts) (Fig. 5). The principal taxa were *P. priestleyi* (10%), *Microcoleus* (5%), and *Pseudanabaena* (4%) and five *Cyanobacteriota* MAGs (Table 1). WG MAG 6 (*Coleofasciculaceae*) and WG MAG 13 (*Phormidesmis*) contained highly complete photosynthetic gene sets with 49 and 45 genes, respectively, and WG MAG 2 (*Microcoleus*) showed the highest expression of *PsaA* and *PsbA*. Conversely, only one MAG from Johnsons Glacier contained *psaA* or *psbA* (JG MAG 14 – *Pelobium*) (Fig. 4).

Evidence for phototrophy was accompanied by strong signatures of autotrophic carbon fixation, particularly via the Calvin–Benson cycle. Genes for this pathway, including *rbcL*, were far more abundant in White Glacier (62.32 cpm) than Johnsons Glacier (69.87 cpm) and were highly expressed in the White Glacier metatranscriptome (824.19 tpm), with nearly 70% of transcripts attributed to *Cyanobacteriota* (Figs 3 and 4). The White Glacier metagenome and metatranscriptome datasets contained nearly complete Calvin–Benson cycle pathways, and *RbcL* was expressed across all *Cyanobacteriota* MAGs, confirming active carbon fixation *in situ*. Eight White Glacier MAGs, five of them *Cyanobacteriota*, and five Johnsons Glacier MAGs encoded *rbcL*.

Additional carbon fixation pathways recovered from both glaciers include the Wood–Ljungdahl pathway (*acsB*), typically associated with anaerobic bacteria and archaea, which was detected at low levels in both glaciers (White Glacier: 3.28 cpm; Johnsons Glacier: 1.23 cpm), while the 3-hydroxypropionate cycle (*mcr*), used by some *Chloroflexota* and *Gammaproteobacteria* [68], was also detected in both glaciers (White Glacier: 45.92 cpm; Johnsons Glacier: 18.39 cpm) and in WG MAG 9 (*Gemmatimonadota*).

*Cyanobacteriota* are well adapted to polar and alpine environments, where they produce exopolysaccharides and cryoprotectants to tolerate freeze–thaw cycles and are widespread in sea ice, cryoconite holes, soils, and glacier margins [69–78]. In White Glacier, they appear to function as the dominant primary producers, as indicated by their prevalence in both gene content and transcriptional activity. The presence and expression of complete photosynthetic and Calvin–Benson cycle pathways in *Cyanobacteriota* MAGs underscore the importance of light-driven primary production in sustaining microbial life in near-surface englacial ice. By supplying fixed carbon and potentially oxygen, *Cyanobacteriota* likely support a broader network of heterotrophic chemolithotrophic taxa, contributing to the greater metabolic diversity observed in White Glacier compared to Johnsons Glacier.

### Active chemolithotrophic pathways contribute to primary production in glacial ice

While the White Glacier englacial ecosystem is primarily dominated by photoautotrophy, lithotrophic metabolisms, including sulfur and nitrogen cycling, also contribute to primary production and energy conservation.

Although sulfate was below the limit of quantification, total sulfur was measurable in both glaciers (White: 0.245 mg/l; Johnsons: 0.059 mg/l; Table 2). Genes for reduced sulfur oxidation were widespread, including *fccA* (sulfide to sulfur; White Glacier: 249.26 cpm; Johnsons Glacier: 306.43 cpm), *sqr* (sulfide to sulfur; White: 68.88 cpm; Johnsons: 258.63 cpm), *soxB* (thiosulfate oxidation; White Glacier: 122.99 cpm; Johnsons Glacier: 89.48 cpm), and *dsrA* (sulfide to sulfite; White Glacier: 1.64 cpm; Johnsons Glacier: 8.58 cpm) (Fig. 4). Marker genes were present in 5 White Glacier MAGs and 13 Johnsons Glacier MAGs, with 3 MAGs; WG MAG 4 (*Armatimonadota*), JG MAG 9 (*Betaproteobacteria*), and JG MAG 16 (*Alphaproteobacteria*) encoding *fccA, sqr*, and *soxB*, suggesting broad substrate use. Several White Glacier MAGs; WG MAG 3 (*Microcoleus*), WG MAG 4 (*Chthonomonadaceae*), WG MAG 9 (*Gemmatimonas*), and WG MAG 11 (*Steroidobacteraceae*) encoded sulfur oxidation genes alongside carbon fixation and respiration, indicating possible facultative chemolithoautotrophy.

Sulfur oxidation genes were actively transcribed in White Glacier, particularly *DsrA* (462.29 tpm) attributed to WG MAG 3 (*Microcoleus*). Although *Microcoleus chthonoplastes* performs sulfur oxidation in hypersaline mats [79, 80], the genus has not previously been observed performing this function in cold or subzero environments. Other transcripts included *FccA* (117.06 tpm), *SoxB* (18.79 tpm), and *Sqr* (16.52 tpm), primarily attributed to *Pseudomonadota* (Fig. 5).

Despite extremely low nitrogen concentrations (often below detection; Table 2), denitrification marker genes were present in both glaciers, with *napA* (nitrate to nitrite; White Glacier: 393.57 cpm; Johnsons Glacier: 311.34 cpm) being the most abundant, while *narG, nirK, norB*, and *nosZ* were also detected (Fig. 4). These genes were distributed across 7 White Glacier MAGs and 12 Johnsons Glacier MAGs, several of which also encode sulfur oxidation pathways (e.g. WG MAG 4, *Chthonomonadaceae*), suggesting potential coupling of sulfur oxidation and denitrification. Many of these MAGs also contained sulfur oxidation pathways, e.g. WG MAG 4 (*Chthonomonadaceae*), indicating potential for coupled sulfur oxidation and denitrification. In White Glacier, *NapA* was also transcribed (71.19 tpm), attributed to *Pseudomonadota* and six other MAGs.

Together, these results indicate that chemolithoautotrophic and photoautotrophic processes jointly support microbial metabolism in glacial ice, with sulfur compounds acting as key electron donors in both glaciers and supporting chemolithoautotrophy in White Glacier. Similar sulfur-driven metabolisms have been reported in sulfur-rich glacial environments such as Borup Fiord Pass, indicating that sulfur cycling may be widespread in surface and englacial ice [81]. The detection of denitrification pathways further suggests activity within anaerobic microenvironments, potentially coupled to sulfur oxidation, as previously observed in sea ice and glacial surface ecosystems [82, 83]. Overall, White Glacier hosts a transcriptionally active and metabolically diverse microbial ecosystem sustained by sulfur oxidation, denitrification, and photosynthesis (Fig. 5).

Although a metatranscriptome was not obtained from Johnsons Glacier, we used iRep analysis as an alternative means of assessing activity which suggested *in situ* metabolic activity of Johnsons Glacier MAGs (Supplemental File). iRep estimates replication rates from coverage variation across a genome and requires high-quality MAGs (≥75% completeness, low fragmentation, ≤2% contamination) for reliable inference [44]. Analyses were therefore limited to MAGs meeting these thresholds, representing ~50% of Johnsons Glacier MAGs and only one from White Glacier. Replication estimates should thus be interpreted cautiously as qualitative indicators rather than precise growth rates. Nevertheless, detection of replication signals in these high-quality MAGs, together with the widespread presence of metabolic pathways comparable to those in White Glacier, supports the inference that at least part of the Johnsons Glacier community may be metabolically active *in situ*.

We also note that biological replication was not feasible for either glacier due to logistical constraints associated with drilling, transporting, and preserving ice cores from remote polar regions. Such limitations are common in englacial microbiology and reflect the challenges of conducting multi-omics analyses on ultralow-biomass samples. Accordingly, our findings should be interpreted as representative of the sampled near-surface ice rather than capturing the full spatial or temporal variability within each glacier. Although the lack of biological replicates limits statistical inference, the consistent marker gene profiles observed across geographically isolated glaciers support the robustness of our datasets. Similar metabolic patterns have also been reported in the Northern Schneeferner Glacier (Germany), where diverse carbon fixation pathways, aerobic metabolism, and anaerobic nitrate/nitrite reduction were detected [20], indicating that meaningful ecological insights can nevertheless be drawn.

### Genes involved in cold adaptation are abundant and expressed in near-surface englacial ice

**Figure 6:**
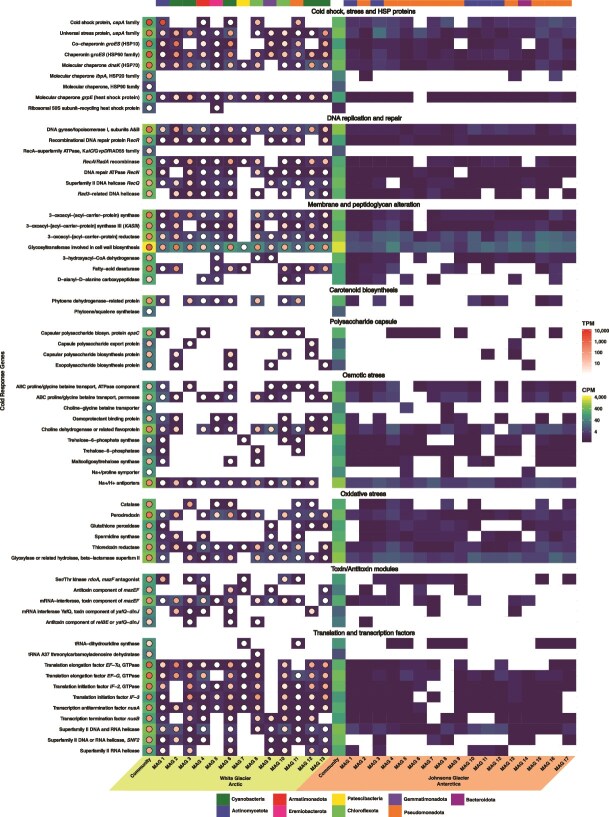
Comparison of genes implicated in adaptation to cold temperatures from the metagenomes, metatranscriptome, and MAGs of White Glacier and Johnsons Glacier; genes in the metagenome and MAGs are represented by the square heatmap and expressed as CPM; the metatranscriptome aligned to the White Glacier metagenome and MAGs is represented by the circle heatmap overlaying the squares and is expressed as the number of TPM; the colors above each column correspond to the phylum of each MAG.

We screened near-surface englacial metagenomes and metatranscriptomes for 61 genes enriched in cold-adapted microorganisms relative to mesophiles, spanning functions such as cold shock response, DNA repair, membrane and peptidoglycan modification, carotenoid and polysaccharide biosynthesis, osmotic and oxidative stress tolerance, toxin–antitoxin modules, and transcription/translation factors [84]. Genes from all categories were present and expressed (White Glacier: 19 778.52 cpm; Johnsons Glacier: 17 976.63 cpm), indicating broad adaptation to cold conditions. Broad overlap in the presence/absence of categories between the glacier communities again suggests similar near-surface ice adaptive potential despite taxonomic differences (Fig. 6).

In both glaciers, “membrane and peptidoglycan alteration” and “oxidative stress” genes were most abundant, while “capsule biosynthesis” and “toxin/antitoxin modules” were least abundant. In contrast, transcript data from White Glacier highlighted “cold shock and stress-related proteins” (avg. 1135.23 tpm) and “membrane and peptidoglycan alteration” (avg. 427.85 tpm) as the most actively expressed. Johnsons Glacier MAGs encoded ~3× more osmotic and oxidative stress genes than White Glacier MAGs, possibly reflecting its proximity to the Southern Ocean and recruitment of marine osmotolerant taxa.

### Viable cells from polar near-surface englacial ice show adaptation to their environment

To complement metatranscriptome evidence of microbial activity, we assessed cell viability using culturing and flow cytometry with live/dead staining. Flow cytometry was unsuccessful for Johnsons Glacier but revealed 4.75 × 10^4^ total cells/ml in White Glacier, of which 0.5% (2.4 × 10^2^ cells/ml) stained as live (Table 2). This proportion is consistent with our expectations for such an extreme environment but lower than the values of metabolically active cells reported for sea ice (0.5%–4%) [24], the only other reported estimate from ice we found. This difference likely reflects harsher conditions in glacial ice such as colder temperatures, lower water availability and nutrients, and longer entrapment compared to the seasonality of sea ice [8].

Despite the lack of flow cytometry data from Johnsons Glacier, both glaciers yielded viable isolates: 50 from Johnsons Glacier and 8 from White Glacier (Supplemental File). Roughly half could not grow above 15°C, and all tested isolates metabolized on Biolog plates at −2°C (Supplemental File), indicating that many are true psychrophiles. Sixteen isolates also tolerated highly acidic conditions (pH 3–4), consistent with survival in englacial liquid veins that can reach pH 3 [85]. Salinity tolerance was also demonstrated, with 16 isolates capable of growth between 6% and 12% salt. Given the dominance of *Cyanobacteriota* in White Glacier, we also attempted to isolate them using BG11 media but were unsuccessful.

There are important implications for global biogeochemical cycling associated with the identification of active and viable microbial ecosystems within glacial ice. Glaciers and ice sheets cover nearly 10% of Earth’s surface, and their retreat due to global warming will release metabolically active microbial communities into downstream ecosystems. Particularly, the discharge of metabolically active photo and chemolithoautotrophs could expedite soil development in recently deglaciated landscapes, or in marine systems, promote bloom formation, and enhance the transport of organic carbon to depth.

### Glacial ice as an analog for microbial habitability on Mars or the icy moons

Our study demonstrated that near-surface englacial ice hosts active microbial ecosystems (Fig. 5) that hypothetically could have supported extent or extinct microorganisms in similar surface ice environments on Mars and the icy moons of the outer solar system, including active autotrophic metabolisms at subzero and ultra-oligotrophic ambient conditions. On Europa and Enceladus, thin layers of surface ice (20 cm) are sufficient to block harmful gamma radiation from reaching putative englacial habitats below [86] and, as on Earth, nutrients would likely be concentrated in liquid veins as has been modeled in deep Europan ice [87], providing potential sources of energy and water. Within the upper meter of White Glacier, we identified abundant oxygenic and anoxygenic photosynthetic metabolic pathways, as well as carbon fixation via the Calvin–Benson cycle, used by phototrophs. A recent model suggests photosynthesis is plausible within the top meter of Martian mid-latitude ice [88]. Furthermore, Mars’ CO_2_-rich atmosphere (95%) [89] would provide an abundant carbon source for fixation. The discovery of active anoxygenic photosynthesis at subzero temperatures is also intriguing, as it can utilize alternative electron donors like ferrous iron, hydrogen, and sulfide which are present on Mars [90–92].

We also detected active lithoautotrophic metabolic pathways in polar englacial ice that are relevant to Mars and the icy moons. Genes for the Wood–Ljungdahl and 3-hydroxypropionate cycles were detected, both of which have been proposed as likely carbon fixation pathways on Enceladus and utilize HCO_3_^−^, which is probably favored over CO_2_ in its slightly alkaline ocean [93]. We also recovered genes for sulfide and thiosulfate oxidation and sulfate reduction in the metagenomes from both glaciers, with transcriptional activity in White Glacier. Sulfur is abundant on Mars primarily as sulfate [94], while sulfides have also been detected in Gale Crater [90]; sulfate is highly enriched in Europa’s surface ice through exchange with its subsurface ocean and material from Io [95, 96]. The incorporation of reduced sulfur into icy environments suggests that sulfur-based lithoautotrophy may be capable of supporting microbial life under low-oxygen or anoxic conditions, serving as an electron acceptor.

Metabolism of trace gasses has been proposed as a potential microbial survival strategy on Mars [97] and discovered in cold, nutrient-limited polar environments on Earth [55]. Mars’ atmosphere contains trace levels of carbon monoxide (0.058% CO) [98], providing a potential energy source. In englacial ice, genes encoding aerobic CO oxidation were abundant and transcriptionally active, demonstrating that CO metabolism can function in frozen, energy-limited, and microaerophilic conditions. Aerobic respiration was also detected within the polar englacial ice. The Martian atmosphere contains 0.16% O_2_ [98], while radiolysis in Europa’s and Enceladus’ near-surface ice may generate 1.2 × 10^13^ – 1.8 × 10^17^ kg O_2_ [99].

## Conclusion

Our results demonstrate that polar near-surface englacial ice hosts a very low-biomass yet metabolically diverse and active microbial ecosystem. Despite community taxonomic differences between White and Johnsons Glaciers, we observed substantial overlap in their functional potential, including aerobic respiration, carbon monoxide oxidation, oxidation of reduced sulfur, and nitrate reduction. In White Glacier, a diverse, active microbial population performing aerobic respiration and carbon monoxide oxidation is supported by *Cyanobacteriota* performing oxygenic photosynthesis and carbon fixation and other lithoautotrophic taxa fixing carbon and generating energy from reduced sulfur and nitrate. The presence and expression of cold adaptation genes and viable, cold-tolerant isolates support the persistence of life in glacial ice. This work represents the first functional characterization of active microbial life in glacial ice, offering new insights into microbial survival in one of Earth’s most extreme environments. These discoveries broaden our knowledge of microbial resilience in cryoenvironments and provide the foundation for modeling the effects that glacial microbial communities will have on downstream terrestrial and marine environments upon release due to global warming-induced glacial melt. Additionally, the discovery of active metabolisms in ice, relevant to Mars and the icy moons increases the potential habitability of ice on these worlds.

## Supplementary Material

ycag105_Supplementary_material

## Data Availability

Sequencing reads, metagenomes, and MAGs are in NCBI under BioProject PRJNA1335554. JGI metagenome annotations are available under GOLD Study ID Gs0161447.
