# Supplementary material for: Bipolar investigation of near-surface glacial ice reveals an active microbial ecosystem driven by photosynthesis and chemolithoautotrophy
Source: ISME Commun. 2026 Apr 22;6(1):ycag105. doi: 10.1093/ismeco/ycag105 (PMC13184969; doi:10.1093/ismeco/ycag105)
Supplement: ycag105_Supplementary_material [file ycag105_supplementary_material.zip › Supplementary Materials Final Revisions1.docx]

**Supplementary Materials for O’Connor et al. “****Bipolar investigation of near-surface glacial ice reveals an active microbial ecosystem driven by photosynthesis and chemolithoautotrophy”.**

**Extended Materials and Methods**

i. Site description and sample collection

White Glacier is a 38.7 km^2^ valley glacier located ~8 km inland from Expedition Fiord on Axel Heiberg Island in the Canadian High Arctic (79.437380N 90.641717W). The glacier has an altitude of 100 to 1782 m above sea level [1]. The ice thickness is, on average, 200 m but can exceed 400 m in some locations. The glacier has the longest mass balance record of any alpine glacier in the Canadian Arctic (65 years) and is a world reference glacier within the Global Terrestrial Network of Glaciers. The area around White Glacier is a polar desert, receiving 58 mm of precipitation per year at sea level and 370 mm of precipitation per year at 2120 m [2]. The closest location where mean annual temperature data is available is at the Eureka weather station (~100km east), which has a mean annual temperature of -19.7°C.

Johnsons Glacier is located on Livingston Island in the South Shetland Islands of Antarctica near the Juan Carlos l Spanish Antarctic Station (62.67057S 60.36961W). It is a sea terminating glacier of approximately 5 km^2^ with an altitude between 50 and 330 m a.s.l. [3]. The glacier’s mass balance has been continually monitored since the 2001/2002 field season, one of the region’s longest mass/balance records [3]. As such, it has been included as a benchmark glacier within the Global Terrestrial Network of Glaciers. The average ice thickness of the glacier is 93 m, with a maximum thickness of 160 m [4–6]. The glacier also receives sporadic inputs of volcanic ash deposited from the Deception Island Volcano (Furdada et al., 1999), located approximately 35 km away, with the last eruption occurring in 1970, which covered the eastern portion of the island [7]. Ninety percent of the island is covered in snow and has a mean annual air temperature of -1.5°C.

Using a Kovacs 9 cm corer, from White Glacier, we collected surface one-meter cores, and from Johnsons Glacier, we collected surface cores up to a depth of 1.5 meters. The specific depths chosen for study (0.7 - 0.9 m for White Glacier, 1.0 - 1.2 m for Johnsons Glacier) were chosen because they were deep enough to ensure separation from the surface ice while still allowing the retrieval of a replicate sample from the same depth. On the glaciers, the cores were cut into 20 cm subsections, stored in sterile whirl-pak bags, and transported frozen back to McGill University. An IM150 light meter (Illuminati Instruments, Santa Clara, California, USA) was also lowered into the White Glacier core borehole and covered. Light (LUX) and colour temperature measurements were recorded to estimate the percent of light penetration into the ice. The temperature of the ice was measured immediately after the cores were removed from the borehole by pressing the probe of a thermometer against the inside surface of the borehole.

For measurement of nutrients and metals in the ice, the exterior 5 mm of the core subsections were removed and part of the remaining core was melted and sent for to the University of Alberta Natural Resources Analytical Laboratory for quantification of dissolved metals (Al, Ag, As, B, Ba, Be, Ca, Cd, Co, Cr, Cu, Fe, K, Li, Mg, Mn, Mo, Na, Ni, P, Pb, S, Sb, Se, Si, Sr, Ti, Tl, V, Zn), anions (NH_4_^+^, NO_2_^-^, NO_3_^-^_,_ total organic nitrogen, PO_4_^3-^, SO_4_^2-^, Cl^-^), total carbon, total organic carbon, pH, and electrical conductivity (Supplemental File). Detailed methods for the analysis of dissolved metals, anions, carbon, pH and electrical conductivity can be found at <https://nral.ualberta.ca/analytical-methods/>. But briefly, total organic carbon (TOC), total inorganic carbon (TIC), and total nitrogen (TN) were quantified using a combustion-based TOC analyzer. Samples were acidified and sparged to remove inorganic carbon, with remaining organic carbon oxidized to CO_2_ and detected by non-dispersive infrared detector. Nitrogen compounds were combusted to NO/NO_2_ and measured by chemiluminescence. Anions (NH_4_^+^, NO_2_^-^, NO_3_^-^_,_ PO_4_^3-^, SO_4_^2-^, Cl^-^) were quantified using a Thermo Gallery Plus Autoanalyzer (Waltham, Massachusetts). Anions were detected by standard colourimetric reactions forming coloured complexes, with absorbance measured at anion specific wavelengths. Methods included the salicylate-hypochlorite (NH_4_^+^), ferrithiocyanate (Cl^-^), molybdenum blue (PO_4_^3-^), hydrazine/vanadium reduction (NO_2_^-^ + NO_3_^-^), and barium chloride turbidimetric (SO_4_^2-^) assays. Dissolved metals, sulfur and phosphorus were measured using ICP-OES (Thermo iCAP6300 Duo, Waltham, Massachusetts). Samples were nebulized into an argon plasma, atomized, and excited to produce element specific emission lines, which were detected by spectrometry. Calibration of all elements and anions was performed using certified standards.

ii. Core decontamination and processing

A negative control artificial core was constructed by freezing double-filtered milli-Q water (18.2 MΩ cm, 25°C) in a whirl-pak into the shape of a cylinder. This artificial core was melted, filtered, and processed the same way as the White Glacier and Johnsons Glacier cores. Adapting the method described by [8], all tools and surfaces were cleaned with DNAse, Rnase, 70% ethanol, and UV sterilized for 30 minutes prior to contacting the core. In a biological safety cabinet, the exterior 5 mm of the core subsections were removed using a saw and then washed with 70% ethanol. The decontaminated core was added to a sterile whirl-pak bag with an equal volume (~1 litre) of DNA/RNA Shield (Zymo Research, Irvine, California) and left to thaw in the dark at 4°C. In total, it took approximately 16 hours for the cores to melt. In this way, as soon as microbial cells melted from the ice, they would be preserved, thus halting all metabolism and preserving the *in situ* metatranscriptome and metagenome. Furthermore, by melting the ice at 4°C instead of room temperature, we hoped to significantly slow any microbial metabolism that might otherwise occur during thaw. Decontamination and processing of the White Glacier core was performed at McGill University, and in the laboratory facilities at the Juan Carlos l Antarctic base for the Johnsons Glacier core.

Once the core subsections had thawed, they were filtered onto a 0.22 µm nitrocellulose membrane. The membranes were then submerged in 5 ml of DNA/RNA Shield and vortexed for 2 minutes with 3 mm sterile DNA/RNA free glass beads to dislodge cells from the membranes. The 5 ml of DNA/RNA Shield reagent containing the cells was then passed through a 50 kDa Amicon® Ultra Centrifugal Filter (Millipore, Burlington, Massachusetts) to further concentrate cells into approximately 800 µl.

iii. DNA/RNA extraction and sequencing

400 µL of the final concentrated cell volume after Amicon filtration of both the artificial core and glacial ice cores were extracted using the ZymoBIOMICS^TM^ DNA/RNA Miniprep Kit (Zymo Research, Irvine, California) using the dual DNA and RNA purification protocol and reducing the final elution volume from 100 µl to 50 µl. The other 400 µL from the Amicon filtrations was saved in case a second extraction was required. 400 µl of Ambion^TM^ Nuclease-Free Water (Invitrogen, Waltham, Massachusetts) was also extracted and used as a negative extraction control. Cleanup of the extractions was performed using the NEB Monarch^®^ DNA and RNA Cleanup Kits (New England Biolabs, Ipswich, Massachusetts), and DNA carryover was removed from the RNA extractions using the Turbo DNA Free Kit (Invitrogen, Waltham, Massachusetts). The DNA and RNA concentration of the sample and negative controls was measured on a Qubit^TM^ 4 Fluorometer using the Qubit^TM^ 1X dsDNA High Sensitivity and RNA High Sensitivity Assay Kits (Invitrogen, Waltham, Massachusetts). DNA was also verified to be absent from the RNA extractions. The RNA extractions’ RNA Integrity Number (RIN) was measured on a Bioanalyzer 2100 using the RNA Pico Kit (Agilent Technologies, Santa Clara, California).

Metagenome library preparation of the samples and negative controls was performed using Illumina’s Nextera XT DNA Library Preparation Kit. The metatranscriptome library preparation was performed using the NEBNext rRNA Depletion and Ultra ll Directional RNA Library Prep Kits and protocol for Illumina (New England Biolabs, Ipswich, Massachusetts). The protocol was modified to avoid the RNA fragmentation step because the RNA integrity number (RIN) values indicated that the RNA did not require further fragmentation. The metagenome and metatranscriptome from White Glacier was sequenced on a NovaSeq 6000 (Illumina, San Diego, California) SP flowcell (2 x 100 base pairs) while the metagenome and metatranscriptome from Johnsosn Glacier was sequenced on a NovaSeq X (Illumina, San Diego, California) 10B flowcell (2 x 150 base pairs), both at The Centre for Applied Genomics at the SickKids Hospital in Toronto, Ontario, Canada.

iv. Metagenome and metatranscriptome data analysis

Low-quality bases, reads, and adapters were trimmed from both the metagenome and metatranscriptome with Trimmomatic (v.0.33, settings LEADING:3 TRAILING:3 SLIDINGWINDOW:4:15) [9]. To remove contaminating sequences, the metagenome reads from the artificial ice core and the negative extraction control were co-assembled using MegaHit (v.1.2.9, setting meta-sensitive) [10] and DeconSeq (v.0.4.3) [11] was used to create a database of these contaminant sequences. DeconSeq was then used to remove sequences from both glacier metagenomes and metatranscriptomes, which mapped to the negative control co-assembly. The decontaminated metagenome reads were taxonomically classified with Kaiju (v.1.9) [12] through the KBase platform [13] then assembled using MegaHit [10]. Genome binning was performed with MetaBat2 (v.2.15) [14], MaxBin2 (v.2.2.7) [15], and SemiBin2 (v.1.5.1) [16]. dRep (v.3.4.3) [17] was used to dereplicate the bins we generated using default parameters. In a small number of bins this resulted in shared contigs among the dereplicated genomes. These shared contigs were removed from the final bin assemblies. We considered a high-quality bin to be >90% complete and <5% contaminated and a medium-quality bin to be >50% and less than 10% contaminated. The completeness and contamination percent of each bin were estimated with CheckM2 (v.1.0.0) [18] and taxonomically identified with GTDB-tk (v.2.2.4) [19]. iRep (v.1.10) [20] was used to estimate the *in situ* replication rate of the microbial population each bin represented. SortMeRNA (v.4.3.6) [21] was used to remove contaminating rRNA sequences from the metatranscriptome and the removehuman tool, available with the BBMap package (v.38.92) (Bushnell B. – sourceforge.net/projects/bbmap/) was used to remove contaminating human DNA from the metatranscriptome. The Metatranscriptome was aligned to the metagenome using bowtie2 (v.2.5.1) [22] and counted using HTSeq2 (v.2.0.2) [23]. Functional annotation was performed by uploading the metagenomes to the JGI IMG/M annotation pipeline [24].

v. Ice core cell isolation and characterization

From each glacier, a replicate core from the same depth used for sequencing was decontaminated as stated above, and 500 ml was melted at 4°C in the dark. The melted ice was filtered onto a 0.22 µm nitrocellulose membrane. The microorganisms from the membrane were then resuspended in 10 ml of PBS buffer and plated on various agar media and enrichment broths. Specifically, resuspended cells were plated on R2A, 1/10 R2A, 1/100 R2A, R2B, ¼ R2B, 1/10 R2B, TSA, 1/10 TSA, 1/100 TSA and in broth enrichments of R2A, ¼ R2A, 1/10 R2A, R2B, ¼ R2B, 1/10 R2B, TSB, 1/10 TSB, and 1/100 TSB. Plates were incubated at 5°C for four months, and enrichment cultures were incubated at 5°C for six months with continual shaking before plating on equivalent agar plates. To recover Cyanobacteriota isolates, 375 ml of the decontaminated core was melted overnight at 10°C. The melted ice was filtered through a 0.22 µm PES membrane filter (Millipore, Burlington, Massachusetts, USA). The filter was placed in 5 ml of liquid BG11 media and vortexed to resuspend cells. 100 µl of the solution was used to inoculate BG11 agar plates and 25 ml of liquid BG11 in triplicate and duplicate, respectively. The cultures were incubated at 5 and 15 °C under light (60-70 µmol//m^2^) for 6 weeks. Colonies isolated from plates were then further tested for growth at 37°C, 25°C, 15°C, 10°C, -2°C, and -5°C. Colonies were also tested for growth on plates incubated at 5°C, supplemented with 1%, 3%, 6%, 9%, 12%, 15%, and 18% NaCl, and on plates incubated at 5°C and adjusted to pH 11, pH 10, pH 9, pH 8, pH 7, pH 6, pH 5, pH 4, pH 3. All colonies isolated from White Glacier and 14 randomly selected colonies isolated from Johnsons Glacier also had their metabolism characterized using Biolog Gen lll plates (Biolog, California, USA). To do this, cell cultures were diluted to a 95% turbidity in IF-C buffer (Biolog, California, USA), and 100 µl was inoculated into each well of the plates. Absorbance changes were measured weekly at a wavelength of 595 nm with a SpectraMax M2e Multi-Mode Microplate Reader (Molecular Devices, San Jose, California, USA). Positive substrate utilization were determined once the Average Well Colour Development (AWCD) of the plate had plateaued [25]. Substrate utilization was considered positive if its OD_595_ value reached above 0.25 [26, 27].

Sanger sequencing was attempted on some isolates to determine their identity; however, it was only successful for some isolates. First, isolated colonies were lysed by mixing with 250 mL of deionized H2O and heating in a microwave for 3 min. Polymerase chain reaction (PCR) amplification of the 16S rRNA gene was performed with primers 27F (5’-AGRGTTTGATCMTGGCTCAG-3’) and 1492R (5’-GGTTACCTTGTTACGACTT-3’), which amplify nearly the full length of the gene (~1500 bp). The 16S rRNA PCR cycling conditions were as follows: (1) 95°C for 7min, (2) 94°C for 45 s, (3) 55°C for 45 s, (4) 72°C for 1min (where steps 2–4 were repeated 30 times), (5) 72°C for 10 min. Amplicon DNA was sent to the Plate-forme d’Analyses Genomiques de l’Universite´ Laval (Quebec City, Quebec, Canada) for sequencing. Low-quality sequences were trimmed, and miscalled bases corrected, with 4Peaks v. 1.8. Sequences were then compared against the GenBank database by using BLASTn to look for the most similar sequences to the query sequences.

vi. Flow cytometry

To quantify the total and live cell concentration in the ice core, an aliquot of resuspended cells in PBS was live/dead stained using the LIVE/DEAD^TM^ BacLight^TM^ Bacterial Viability Kit (Invitrogen, Waltham, Massachusetts) following the manufacturer’s instructions. One of two 400 µL aliquots of resuspended cells was left unstained, and 1.5 µL of SYTO® 9 and propidium iodide were added to the other. Flow cytometry measurements were conducted on a Guava easyCyte (Millipore, Burlington, Massachusetts) after samples were incubated for 15 minutes at room temperature in the dark. The adjustments made to the gains in the red and green fluorescent channels, forward and side scatter, and live and unstained (boiled for 10 minutes) culture controls were made to ensure that most of the data points were within the dynamic range. Based on controls, the regions were classified as live and dead. The sample was subjected to 5000 events, and the concentration of viable bacteria was determined by subtracting the blank value.

**Commentary on metatranscriptome data presented in this manuscript**

i. Absence of a metatranscriptome from Johnsons Glacier

Metatranscriptome sequencing from Johnsons Glacier was not successful, potentially for several reasons as follows. Firstly, melting and filtering of the ice was performed at the Juan Carlos l research station in Antarctica. While the filters were preserved in DNA/RNA Shield, the cold chain could not be maintained for the trip back to our laboratory, which could have resulted in the degradation of the RNA. Secondly, when preparing RNA libraries from low biomass samples it is very easy to lose the genetic material due to small pipetting errors or because library protocols are not optimized for such low inputs of RNA. Thirdly, transcription may have been present but too low to detect. Finally, we cannot discount the possibility that there is no active in situ microbial community in Johnsons Glacier, although this would be surprising given that the glacier contains an abundant viable subzero microbial community and iRep analysis suggests that metagenome assembled genomes (MAGs) from Johnsons Glacier were performing cell division when the ice was collected.

ii. Possibility that the metatranscriptome presented from White Glacier does not result from in situ metabolism

There is the possibility that the metatranscriptome analyses presented here are not representative of in situ metabolism but rather ex-situ metabolism, either after the ice was collected or during ice melt. The ice cores were kept in the dark after collection, during transport, and while being thawed into DNA/RNA Shield solution, supporting the notion in situ metabolism was present prior to sampling; for example, the relatively large number of transcripts related to photosynthesis rules out the possibility that the transcripts within our dataset resulted from ex-situ metabolism. Furthermore, by melting the ice directly into DNA/RNA Shield, the active microbial community would cease all metabolism upon release from the ice and, if not, then the low temperature the ice was melted at (4°C) would keep levels of ex-situ metabolism negligible.

**References:**

1. Thomson LI, Copland L. Multi-decadal reduction in glacier velocities and mechanisms driving deceleration at polythermal White Glacier, Arctic Canada. *Journal of Glaciology* 2017;**63**:450–463. https://doi.org/10.1017/jog.2017.3.

2. Cogley JG, Adams WP, Ecclestone MA, Jung-Rothenhäusler F, Ommanney CSL. Mass balance of White Glacier, Axel Heiberg Island, N.W.T., Canada, 1960–91. *Journal of Glaciology* 1996;**42**:548–563. https://doi.org/10.3189/s0022143000003531.

3. Navarro FJ, Jonsell UY, Corcuera MI, Martín-Español A. Decelerated mass loss of Hurd and Johnsons Glaciers, Livingston Island, Antarctic Peninsula. *Journal of Glaciology* 2013;**59**:115–128. https://doi.org/10.3189/2013jog12J144.

4. Benjumea B, Macheret YY, Navarro FJ, Teixidó T. Estimation of water content in a temperate glacier from radar and seismic sounding data. *Ann Glaciol* 2003;**37**:317–324. https://doi.org/10.3189/172756403781815924.

5. Navarro FJ, Macheret YY, Benjumea B. Application of radar and seismic methods for the investigation of temperate glaciers. *J Appl Geophy* 2005;**57**:193–211. https://doi.org/10.1016/j.jappgeo.2004.11.002.

6. Navarro FJ, Otero J, Macheret YY, Vasilenko E V., Lapazaran JJ, Ahlstrøm AP, et al. Radioglaciological studies on Hurd Peninsula glaciers, Livingston Island, Antarctica. *Ann Glaciol* 2009;**50**:17–24. https://doi.org/10.3189/172756409789097603.

7. Baker PE, Mcreath I. Investigation of the 1970 volcanic activity at Deception Island. South Sheltlands Islands. *Polar Record* 1972;**16**:67–71. https://doi.org/10.1017/s0032247400062458.

8. Coelho LF, Blais MA, Matveev A, Keller-Costa T, Vincent WF, Costa R, et al. Contamination analysis of Arctic ice samples as planetary field analogs and implications for future life-detection missions to Europa and Enceladus. *Scientific Reports* 2022;**12**:1–13. https://doi.org/10.1038/s41598-022-16370-5.

9. Bolger AM, Lohse M, Usadel B. Trimmomatic: a flexible trimmer for Illumina sequence data. *Bioinformatics* 2014;**30**:2114–2120. https://doi.org/10.1093/bioinformatics/btu170.

10. Li D, Liu C-M, Luo R, Sadakane K, Lam T-W. MEGAHIT: an ultra-fast single-node solution for large and complex metagenomics assembly via succinct de Bruijn graph. *Bioinformatics* 2015;**31**:1674–1676. https://doi.org/10.1093/bioinformatics/btv033.

11. Schmieder R, Edwards R. Fast Identification and Removal of Sequence Contamination from Genomic and Metagenomic Datasets. *PLoS One* 2011;**6**:e17288. https://doi.org/10.1371/journal.pone.0017288.

12. Menzel P, Ng KL, Krogh A. Fast and sensitive taxonomic classification for metagenomics with Kaiju. *Nature Communications* 2016;**7**:1–9. https://doi.org/10.1038/ncomms11257.

13. Arkin AP, Cottingham RW, Henry CS, Harris NL, Stevens RL, Maslov S, et al. KBase: The United States Department of Energy Systems Biology Knowledgebase. *Nature Biotechnology* 2018;**36**:566–569. https://doi.org/10.1038/nbt.4163.

14. Kang DD, Li F, Kirton E, Thomas A, Egan R, An H, et al. MetaBAT 2: An adaptive binning algorithm for robust and efficient genome reconstruction from metagenome assemblies. *PeerJ* 2019;**2019**. https://doi.org/10.7717/peerj.7359.

15. Wu YW, Simmons BA, Singer SW. MaxBin 2.0: an automated binning algorithm to recover genomes from multiple metagenomic datasets. *Bioinformatics* 2016;**32**:605–607. https://doi.org/10.1093/bioinformatics/btv638.

16. Pan S, Zhao XM, Coelho LP. SemiBin2: self-supervised contrastive learning leads to better MAGs for short- and long-read sequencing. *Bioinformatics* 2023;**39**:i21–i29. https://doi.org/10.1093/bioinformatics/btad209.

17. Olm MR, Brown CT, Brooks B, Banfield JF. dRep: a tool for fast and accurate genomic comparisons that enables improved genome recovery from metagenomes through de-replication. *ISME J* 2017;**11**:2864–2868. https://doi.org/10.1038/ismej.2017.126.

18. Chklovski A, Parks DH, Woodcroft BJ, Tyson GW. CheckM2: a rapid, scalable and accurate tool for assessing microbial genome quality using machine learning. *Nat Methods* 2023;**20**:1203–1212. https://doi.org/10.1038/s41592-023-01940-w.

19. Chaumeil P-A, Mussig AJ, Hugenholtz P, Parks DH. GTDB-Tk: a toolkit to classify genomes with the Genome Taxonomy Database. *Bioinformatics* 2020;**36**:1925–1927. https://doi.org/10.1093/bioinformatics/btz848.

20. Brown CT, Olm MR, Thomas BC, Banfield JF. Measurement of bacterial replication rates in microbial communities. *Nature Biotechnology 2016 34:12* 2016;**34**:1256–1263. https://doi.org/10.1038/nbt.3704.

21. Kopylova E, Noé L, Touzet H. SortMeRNA: fast and accurate filtering of ribosomal RNAs in metatranscriptomic data. *Bioinformatics* 2012;**28**:3211–3217. https://doi.org/10.1093/bioinformatics/bts611.

22. Langmead B, Salzberg SL. Fast gapped-read alignment with Bowtie 2. *Nature Methods* 2012;**9**:357–359. https://doi.org/10.1038/nmeth.1923.

23. Putri GH, Anders S, Pyl PT, Pimanda JE, Zanini F. Analysing high-throughput sequencing data in Python with HTSeq 2.0. *Bioinformatics* 2022;**38**:2943–2945. https://doi.org/10.1093/bioinformatics/btac166.

24. Chen IMA, Chu K, Palaniappan K, Ratner A, Huang J, Huntemann M, et al. The IMG/M data management and analysis system v.7: content updates and new features. *Nucleic Acids Res* 2023;**51**:D723–D732. https://doi.org/10.1093/nar/gkac976.

25. Garland JL, Mills AL. Classification and characterization of heterotrophic microbial communities on the basis of patterns of community-level sole-carbon-source utilization. *Appl Environ Microbiol* 1991;**57**:2351–9. https://doi.org/10.1128/aem.57.8.2351-2359.1991.

26. Garland JL. Analytical approaches to the characterization of samples of microbial communities using patterns of potential C source utilization. *Soil Biol Biochem* 1996;**28**:213–221. https://doi.org/10.1016/0038-0717(95)00112-3.

27. Garland JL. Analysis and interpretation of community-level physiological profiles in microbial ecology. *FEMS Microbiol Ecol* 1997;**24**:289–300. https://doi.org/10.1111/j.1574-6941.1997.tb00446.x.
